# Application of Artificial Intelligence in the Prediction and Management of Stroke Rehabilitation

**DOI:** 10.34133/cbsystems.0702

**Published:** 2026-10-08

**Authors:** Weihua Song, Shuai Si, Yinghui Wang, Hong Chang, Xiaoying Tang

**Affiliations:** ^1^ Department of Neurology, Beijing Municipal Geriatric Medical Research Center, Xuanwu Hospital, Capital Medical University, Beijing 100053, China.; ^2^ School of Medical Technology, Zhengzhou Academy of Intelligent Technology, Beijing Institute of Technology, Beijing 100081, China.; ^3^ Luohe Orthopedic Hospital, Luohe 462001, China.

## Abstract

Artificial intelligence is being studied across the stroke care pathway, but evidence from acute diagnosis, early prognosis, rehabilitation-outcome prediction, and long-term management is often discussed without clearly separating these clinical tasks. This structured narrative review adds an integrated framework that maps each application to its decision point, relevance to rehabilitation, validation level, and readiness for clinical use. Unlike previous technology-centered reviews, it explicitly separates acute prognostic evidence from rehabilitation-specific evidence and distinguishes technical performance from transportability, clinical impact, and rehabilitation benefit. Acute imaging and prognostic models may provide baseline information for later rehabilitation planning, but their diagnostic or prognostic performance does not establish rehabilitation efficacy. Most rehabilitation models and robotic, virtual-reality, brain–computer interface, wearable, and home-monitoring applications remain supported mainly by internal validation or early exploratory studies. Small or selected datasets, limited external and prospective validation, uncertain workflow effects, and sparse patient-centered outcomes continue to constrain clinical interpretation.

## Introduction

### Importance of stroke rehabilitation and limitations of existing methods

Stroke is a major cause of long-term disability and places sustained pressure on patients, families, and health systems [1]. Although rehabilitation should be individualized and goal-directed, routine decisions still rely heavily on periodic assessment, clinician experience, and local resources, which may not fully reflect differences in baseline function, comorbidities, lesion characteristics, and recovery pace [1].

Rehabilitation delivery and access also vary across health systems [2]. Geographic barriers, limited service capacity, and intermittent outcome assessment can restrict access and delay treatment adjustment. These limitations support the development of more adaptive approaches that can be evaluated consistently across sites [3].

### Application prospect of artificial intelligence in stroke rehabilitation

Artificial intelligence (AI) may support recovery prediction, movement or imaging analysis, monitoring, and treatment adaptation [4–6]. Reported benefits must nevertheless be interpreted in relation to study design, sample size, comparator, outcome definition, follow-up, and validation setting.

In an external scoping-review dataset, impairment and assisted interventions represented 41.6% and 33.8% of articles, respectively, accounting together for 75.4% of the literature [7]. Functional impairment/capacity, brain–computer interfaces (BCIs), and gait and mobility were the most frequent individual topics, whereas prediction, imaging/neuroscience, and motor-function recovery were less represented. A separate bibliometric analysis likewise identified rehabilitation, machine learning (ML), recovery, and upper-limb function as prominent themes [8].

Acute detection, triage, and risk-management tools are relevant because early treatment and severity characterization shape the later care pathway [9,10]. A 2025 conference abstract is mentioned only as contextual evidence outside the 108-study synthesis [11]. Acute diagnostic performance does not establish rehabilitation benefit, and clinical use additionally requires transportability, interpretable outputs, secure data governance, and compatibility with routine workflows.

### Objectives and scope of literature review

This review examines AI at successive decision points along the stroke care pathway instead of cataloguing algorithms or devices in isolation. It covers prediction, assessment, treatment support, monitoring, and rehabilitation management while keeping acute-care tasks separate from rehabilitation-specific applications. The analysis also considers how validation quality, technology integration, and implementation barriers shape the clinical interpretation of findings involving robotics, virtual reality (VR), BCIs, wearables, and telerehabilitation.

The temporal evolution of AI techniques applied to stroke rehabilitation and recovery research is summarized in Fig. 1.

**Fig. 1.**
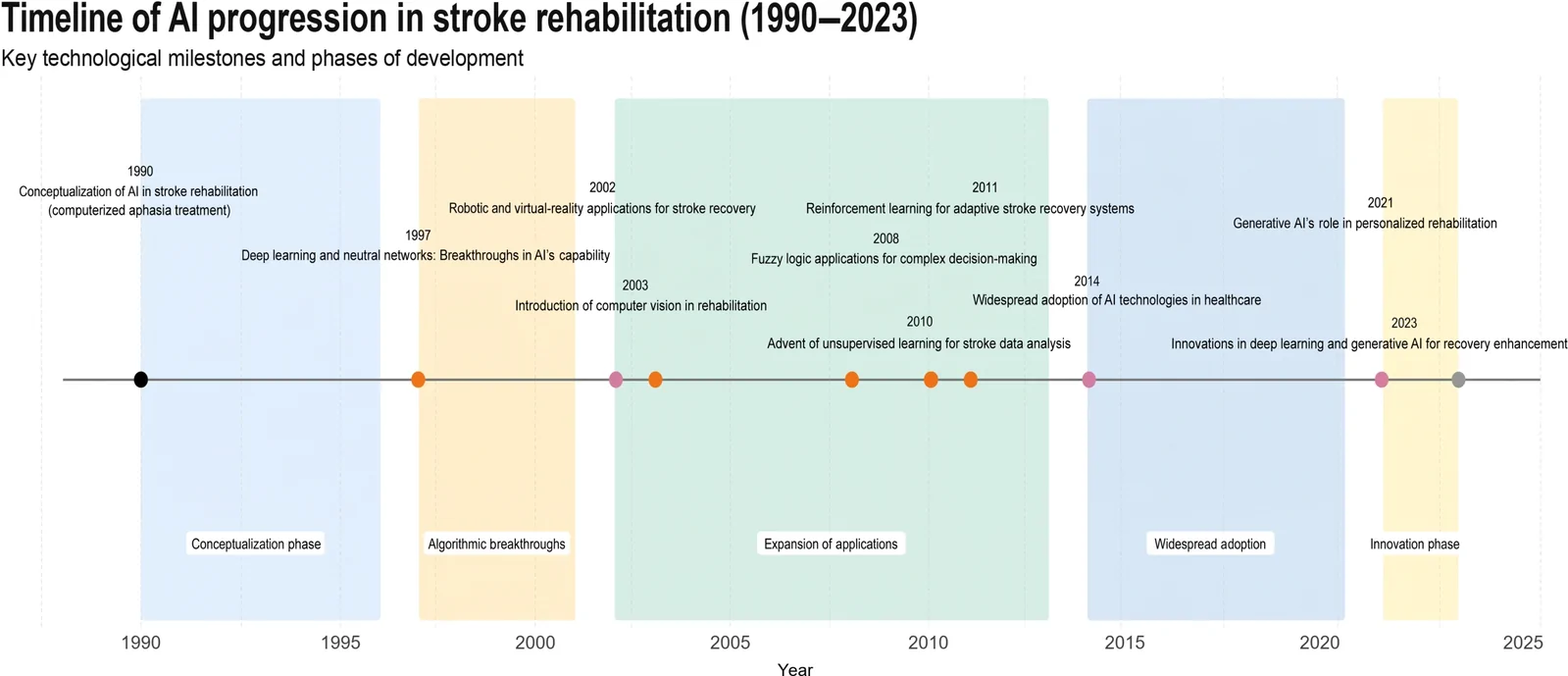
Evolution of artificial intelligence techniques in stroke rehabilitation and recovery research.

The timeline summarizes the transition from conventional ML approaches to deep learning (DL), multimodal modeling, wearable sensing, and adaptive rehabilitation systems discussed in the accompanying text. It includes selected historical milestones before 2010 for context and is therefore not a plot of the January 2010 to March 2025 literature-search results. Source: conceptual figure created by the authors from the literature cited in this review. AI, artificial intelligence.

Regulatory authorization is currently confined mainly to selected acute imaging functions. Evidence for rehabilitation prediction, assessment, and assistive technologies still comes largely from exploratory or early feasibility studies.

## Methodology of the Literature Review

### Review design and information sources

This study was designed as a structured narrative review. A structured, multidatabase literature search and a Preferred Reporting Items for Systematic Reviews and Meta-Analyses (PRISMA)-style description of study identification and selection were employed to improve reporting transparency. The review was not designed or registered as a systematic review, and no meta-analysis was performed.

Four electronic databases were searched: PubMed/MEDLINE, IEEE Xplore, Web of Science, and Scopus. They were selected to cover complementary literature in biomedical science, rehabilitation medicine, engineering, and computer science. The reference lists of key reviews and relevant conference proceedings were also examined to identify supplementary background sources. Because the yield of this supplementary searching was not recorded prospectively, these sources were used only for contextual discussion and were not added to the 108 publications in the database-derived thematic synthesis or to the PRISMA-style selection counts.

### Search strategy

The database searches were conducted between January and March 2025 and covered publications from January 2010 through March 2025. March 2025 represented the date of the final database search and served as a consistent cutoff across all information sources. The 15-year interval from January 2010 through March 2025 was selected to capture the rapid development of ML, DL, and multimodal AI applications in stroke rehabilitation.

The search strategy employed a combination of Medical Subject Headings terms and free-text keywords, organized into 3 conceptual groups connected by Boolean operators. The condition group included terms such as “stroke”, “cerebrovascular accident”, “ischemic stroke”, “hemorrhagic stroke”, and “cerebral infarction”. The technology group included terms such as “artificial intelligence”, “machine learning”, “deep learning”, “neural network”, “natural language processing”, “computer vision”, “brain-computer interface”, “robotics”, “wearable sensors”, “virtual reality”, and “telerehabilitation”. The outcome group included terms such as “rehabilitation”, “recovery”, “prediction”, “prognosis”, “functional outcome”, “motor recovery”, “cognitive rehabilitation”, and “speech therapy”. Terms within each group were combined with the OR operator, and the 3 groups were combined with the AND operator.

### Eligibility criteria

Studies were included if they met the following criteria: (a) peer-reviewed original research articles, review articles, or conference papers published in English; (b) studies investigating the application of AI or ML techniques in any aspect of stroke rehabilitation, including prediction, assessment, intervention, monitoring, or management; (c) studies involving adult stroke patients (aged ≥18 years) or computational models developed for adult stroke rehabilitation; and (d) studies published from January 2010 through March 2025.

Publications were excluded if they (a) focused exclusively on acute stroke diagnosis or detection without relevance to rehabilitation or recovery prediction; (b) were editorials, commentaries, letters to the editor, or abstracts without full-text availability; (c) were not published in English; (d) addressed nonstroke neurological conditions without specific applicability to stroke rehabilitation; or (e) described purely theoretical AI frameworks without clinical validation or rehabilitation-relevant data. Sources cited only to provide regulatory, historical, or postsearch context were not counted among the 108 publications included in the thematic synthesis; when used, their contextual status is stated explicitly.

### Study selection and data extraction

The 4 database searches identified 1,247 records. After 389 duplicates were removed, 858 unique records underwent title and abstract screening. Based on the recorded stage totals, 546 records were excluded during title and abstract screening, and 312 articles proceeded to full-text eligibility assessment. An additional 204 full-text articles were excluded, leaving 108 publications for inclusion in the database-derived narrative synthesis. Two reviewers independently conducted the screening, and disagreements were resolved through discussion and consensus. The study-selection process is summarized in the PRISMA-style flow diagram in Fig. 2. The 108 included publications, and all counts in Fig. 2 derive exclusively from the 4 database searches. Supplementary sources identified from reference lists or conference proceedings were used only for background context and were not included in these counts. Reason-specific counts for full-text exclusions were not available in the screening documentation and therefore were not inferred.

**Fig. 2.**
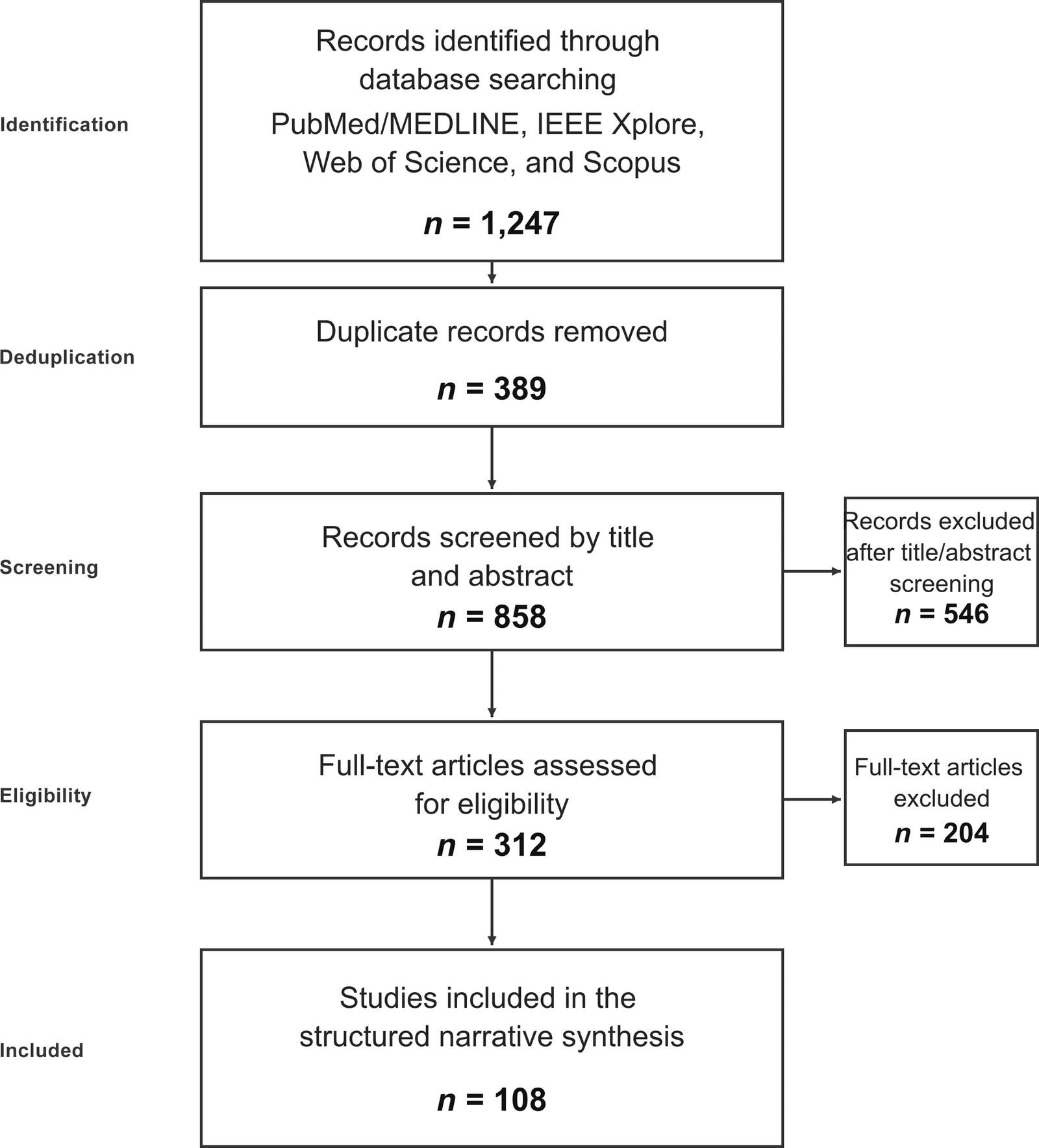
PRISMA-style flow diagram of literature identification, screening, eligibility assessment, and inclusion in the structured narrative review. Source: generated by the authors from the 4-database screening totals reported in the “Study selection and data extraction” section. All counts in the diagram refer exclusively to the database-derived selection process. Supplementary background sources identified from reference lists or conference proceedings were not added to the 108 included publications or to the diagram counts. PRISMA, Preferred Reporting Items for Systematic Reviews and Meta-Analyses.

For each included study, the reviewers extracted the study design, AI technique, sample size, participant characteristics, rehabilitation domain, primary outcome, performance metrics, validation method, and reported limitations.

### Methodological appraisal

Because this structured narrative review included heterogeneous evidence types—original studies, reviews, and conference papers—no single formal risk-of-bias instrument could be applied appropriately and consistently across all sources. The Cochrane Risk of Bias tool and the Newcastle–Ottawa Scale were not used because neither can accommodate the full range of study designs included. The purpose of the review was thematic narrative synthesis rather than pooled effect estimation or formal grading of evidence certainty. Nevertheless, the included literature was appraised qualitatively and systematically with respect to study design, sample size, internal or external validation, performance reporting, clinical relevance, and reported limitations. The absence of a formal study-level quality assessment limits direct comparison of evidence strength across studies and is acknowledged as a limitation of this review.

### Thematic synthesis

The 108 publications included in the present review were organized and synthesized according to 4 thematic domains: (a) AI for prediction of stroke rehabilitation outcomes; (b) AI for rehabilitation assessment and monitoring; (c) AI-driven therapeutic interventions and management systems; and (d) integration of AI with robotics, VR, BCIs, wearable technologies, and telerehabilitation. Within each domain, findings were examined for convergent evidence, methodological strengths and limitations, implementation barriers, and directions for future research.

Five technology-focused articles published after the completion of the March 2025 database search are cited individually in their relevant thematic sections as postsearch contextual updates; they were not included in the 108-publication database-derived synthesis or the PRISMA-style selection counts.

Table 1 is provided as external bibliometric context only. It summarizes the distribution of 704 articles in the independent scoping-review dataset reported by Senadheera et al. [7] and is not a tabulation of the 108 publications included in the present narrative review.

**Table 1.** Article counts of research topics categorized by major research themes in adult stroke recovery and rehabilitation (adapted from Senadheera et al. [7])

Research themes and topics	Articles, *n*	Proportion/%
**Theme 1: Impairment**		
Functional impairment/capacity	115	16.3
Gait and mobility	83	11.8
Electromyography (EMG)/motor impairment	44	6.3
Upper-limb function	28	4.0
Speech	23	3.3
Subtotal: Impairment	293	41.6
**Theme 2: Assisted interventions**		
Brain–computer interface (BCI)	100	14.2
Rehabilitation/robot-assisted therapy	61	8.7
Virtual reality (VR)	33	4.7
Computer vision	23	3.3
Deep-learning-based systems	21	3.0
Subtotal: Assisted interventions	238	33.8
**Theme 3: Prediction**		
Outcome prediction	50	7.1
Analysis for monitoring and service prediction	39	5.5
Motor-function recovery	14	2.0
Subtotal: Prediction	103	14.6
**Theme 4: Imaging and neuroscience**		
Functional connectivity	40	5.7
Medical imaging	30	4.3
Subtotal: Imaging and neuroscience	70	9.9
Total	704	100

Data are adapted from Table 2 of Senadheera et al. [7], a scoping review that included 704 independent articles published up to January 2024, under the Creative Commons Attribution 4.0 International (CC BY 4.0) license. Thus, *N* = 704 refers to the external scoping-review dataset and is separate from the 108 publications included in the present structured narrative review. Theme subtotals and percentages were calculated by the present authors from the source counts using *N* = 704 and are reported to one decimal place; sums of rounded row percentages may differ slightly from 100.0%.

As a structured narrative review, this study remains susceptible to selection bias and does not provide the formal study-level risk-of-bias grading expected of a systematic review. The multidatabase search, explicit eligibility criteria, independent dual-reviewer screening, transparent selection counts, and PRISMA-style flow description were employed to improve reproducibility and clarify the scope of the evidence synthesis. The absence of a formal quality-assessment instrument should be considered when interpreting comparisons across heterogeneous evidence types.

## Basic Concepts and Processes of Stroke Rehabilitation

### Basic concepts of stroke rehabilitation

Stroke rehabilitation integrates motor, cognitive, communication, and psychosocial care to improve function and activities of daily living (ADLs) after cerebrovascular injury. Because motor performance, cognition, language, mood, and social participation interact during recovery, rehabilitation models should account for cross-domain influences rather than treating each impairment as an isolated process. As documented by Langhorne et al. [12], stroke rehabilitation encompasses physical therapy, occupational therapy, and speech–language pathology, which collectively address motor function, self-care, communication, and cognition. Outcomes vary according to baseline impairment, treatment dose, timing, outcome definition, and duration of follow-up. Direct comparisons between rehabilitation modalities require studies with comparable populations, interventions, and endpoints; heterogeneous percentage estimates should not be interpreted as relative treatment effects.

A compartmentalized approach may overlook interactions among motor practice, attention, executive function, and communication. Rehabilitation professionals therefore adapt conventional therapy to each patient’s clinical profile and goals. AI and robotic technologies are being investigated as adjuncts for assessment, feedback, and task practice, but their incremental benefit over clinician-directed rehabilitation remains application specific. Stroke rehabilitation extends beyond motor recovery to include independence in self-care, communication, swallowing, cognition, psychological well-being, and social participation. Brain stem stroke, for example, may produce communication and swallowing impairments that require task-specific multidisciplinary management [13]. Depression, anxiety, family support, access to care, systemic inflammation, and sarcopenia may also influence recovery, although the magnitude of these associations varies across populations and outcome definitions [14]. A central principle of contemporary rehabilitation is to promote experience-dependent neural plasticity through repetitive, goal-directed practice and structured cognitive stimulation. Grefkes and Fink [15] described neuroplasticity as a central mechanism of poststroke recovery. The nervous system can reorganize structurally and functionally after injury, particularly when rehabilitation provides repetitive, task-specific practice. Motor training, cognitive stimulation, environmental enrichment, and psychosocial support may interact; rehabilitation should therefore address recovery as a multidomain process rather than as a series of isolated impairments.

AI-guided rehabilitation may support the behavioral conditions associated with experience-dependent plasticity by adjusting task difficulty, practice dose, and feedback timing in response to a patient’s performance. Robotic systems can deliver reproducible, high-repetition assisted movement, VR tasks can combine goal-directed practice with augmented sensory feedback, and BCI systems can couple detected neural activity to external feedback or assisted movement [15–29]. These closed-loop features provide a plausible link between adaptive training and the recruitment or reorganization of residual sensorimotor networks.

Adaptive algorithms or short-term task gains should not, however, be interpreted as direct evidence of neural reorganization. Mechanistic claims require longitudinal studies that relate training exposure to functional outcomes and to convergent measures such as structural or functional imaging, electroencephalography (EEG), or neurophysiological assessment.

The multidomain concept of stroke rehabilitation described above is summarized in Fig. 3.

**Fig. 3.**
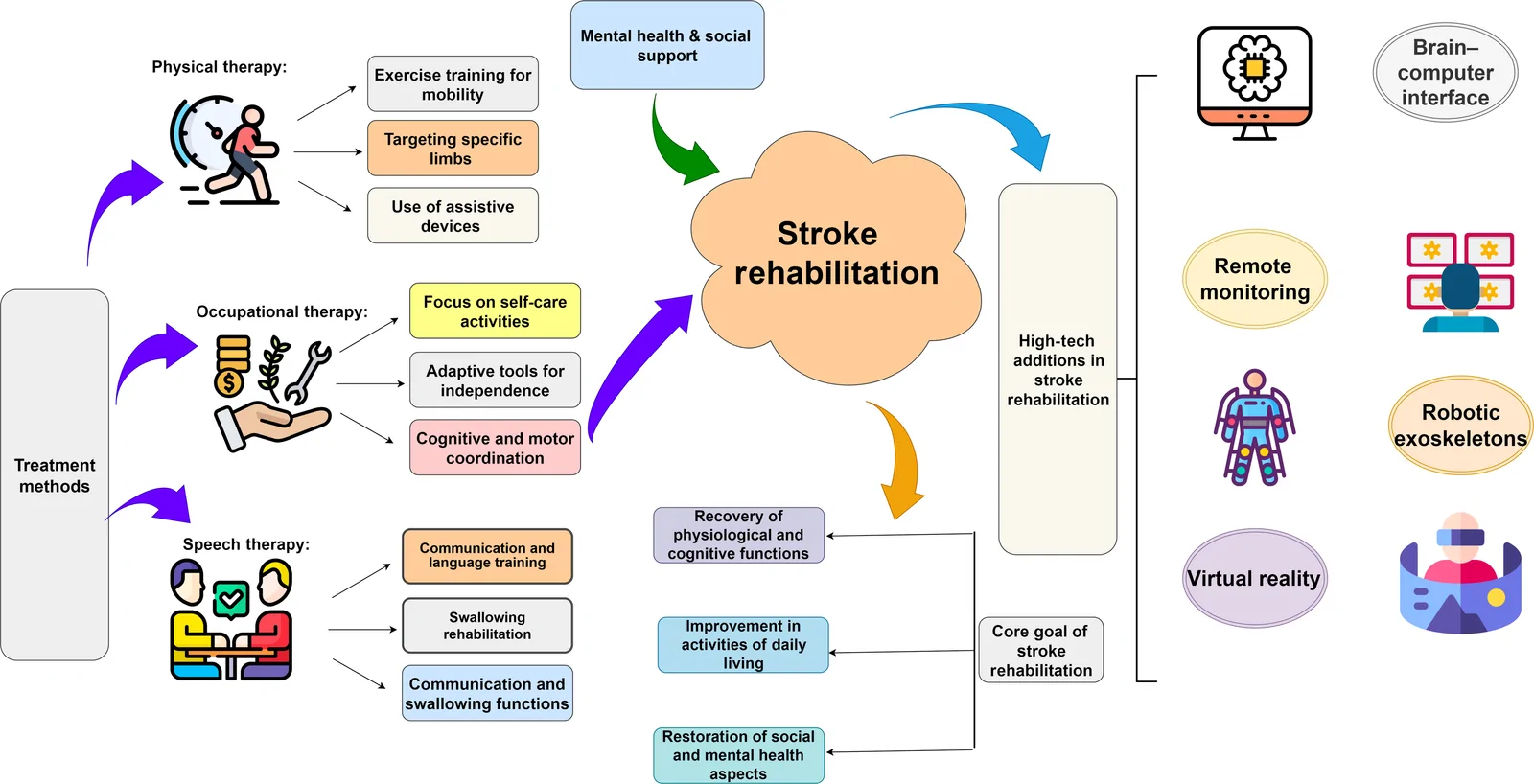
Multidomain framework for stroke rehabilitation.

The figure shows how motor, cognitive, communication, psychological, and participation-related goals interact within individualized rehabilitation. Source: conceptual figure created by the authors.

### Main process of stroke rehabilitation

The 4-stage rehabilitation framework—assessment, treatment, feedback, and adjustment—is best understood as an iterative cycle rather than a strictly linear pathway. Repeated assessment and feedback allow the multidisciplinary team to adapt goals and treatment as neurological status, function, and participation change. During assessment, clinicians evaluate neurological status, motor capacity, cognition, communication, ADLs, and relevant imaging findings. Common instruments include the Fugl–Meyer Assessment, Mini-Mental State Examination, and Barthel Index. These findings establish the baseline for individualized goal setting and treatment planning [30]. Evidence-based quality indicators can support consistent assessment and service evaluation across rehabilitation settings [31].

Coordinated multidisciplinary care is required across these rehabilitation activities [32]. During treatment, physical therapy, occupational therapy, and speech–language pathology address mobility, balance, upper-limb function, self-care, communication, and swallowing according to the patient’s goals and impairments [12,33]. Rehabilitation technologies may supplement clinician-directed care by providing repetitive practice and quantifiable feedback, but technical intensity or device precision should not be equated with superior clinical outcomes [34].

Feedback is obtained from clinical examination, standardized outcome measures, patient reports, and, where appropriate, rehabilitation devices. Kinematic or kinetic measurements can help clinicians identify change and adjust task difficulty, assistance, or practice dose [35]. During the adjustment stage, the team revises goals and treatment parameters in response to observed progress, fatigue, complications, and patient priorities [34]. Together, the 4 stages form a closed-loop process that may be supported—but should not be replaced—by AI. Fig. 4 places potential AI functions within the iterative rehabilitation process. Fig. 4A summarizes long-term poststroke follow-up, referral, and management of rehabilitation needs after discharge [36]. Fig. 4B presents the consensus-based selection of outcome measures for the upper-limb, lower-limb, ADL, stroke-specific, and International Classification of Functioning, Disability and Health domains [37]. Fig. 4C summarizes AI methods and algorithm families relevant to stroke recovery and rehabilitation research [7]. The figure identifies possible points of integration; it does not imply that AI has demonstrated clinical benefit at every stage.

**Fig. 4.**
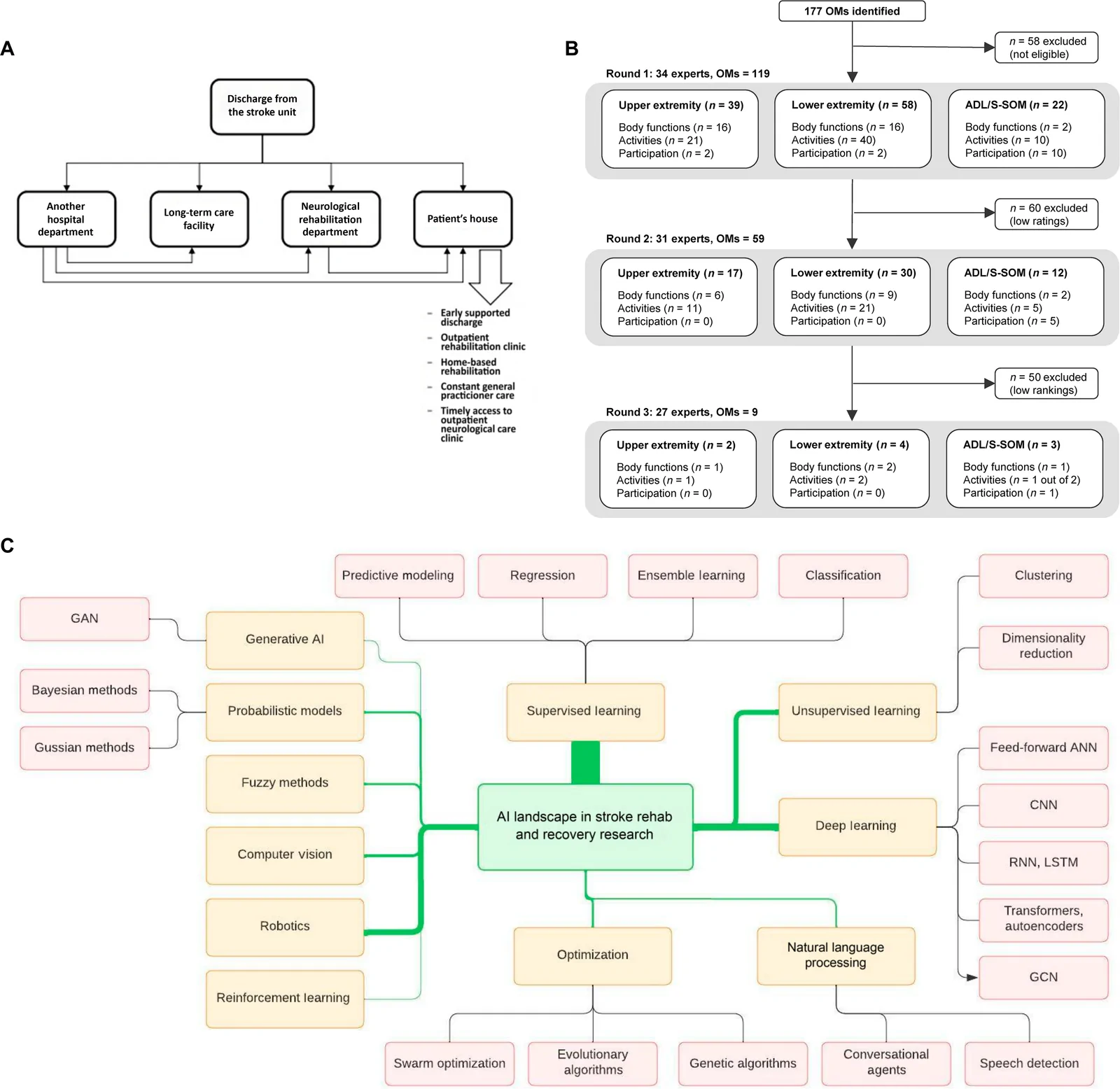
Stroke rehabilitation process and AI intervention nodes. (A) Post-acute-care pathway, including specialist follow-up, referral, and rehabilitation-needs management after discharge [36]. (B) Consensus-based selection of motor-rehabilitation-outcome measures [37]. (C) Overview of AI methods and algorithm families relevant to stroke recovery and rehabilitation research [7]. Stroke rehabilitation is therefore a multidisciplinary process built around assessment, treatment, feedback, and adjustment.Source: adapted from the conceptual frameworks reported by Sarzyńska-Długosz [36], Pohl et al. [37], and Senadheera et al. [7]. ADL, activity of daily living; AI, artificial intelligence; ANN, artificial neural network; CNN, convolutional neural network; GAN, generative adversarial network; GCN, graph convolutional network; LSTM, long short-term memory; OMs, outcome measures; RNN, recurrent neural network; S-SOM, stroke-specific outcome measures.

Traditional rehabilitation principles are well established, but the specific clinical value of AI integration at each intervention node remains incompletely validated.

## Overview of AI Technology

### Basic concepts of AI

AI refers to computational methods that perform tasks involving pattern recognition, prediction, or decision support. Advances in computing, algorithms, and data availability have expanded AI applications in healthcare [38]. In stroke care, these methods are being studied for image analysis, prognostic modeling, rehabilitation assessment, and treatment support; their value depends on the clinical task, data quality, validation design, and effect on patient care. AI methods are applied across the stroke care continuum, but distinct clinical tasks should not be conflated because they use similar computational approaches. Acute diagnosis and imaging triage address large-vessel-occlusion detection, lesion quantification, and emergency treatment selection. Acute or discharge prognostication estimates early functional status, mortality risk, or discharge destination from admission or in-hospital data. Rehabilitation-outcome prediction targets motor recovery, independence in ADLs, participation, or patient-reported outcomes over subsequent weeks or months. Longitudinal monitoring and management address adherence, recurrent-event risk, home-based progress, and service utilization during extended follow-up.

Although these tasks occur along the same care pathway and may use some of the same variables, their targets, time windows, input requirements, decision points, and validation needs differ. Acute imaging characteristics, for example, may enter later rehabilitation models as baseline covariates. Performance on an acute diagnostic or prognostic endpoint cannot be assumed to translate into better rehabilitation treatment or long-term function. That question requires rehabilitation-specific endpoints and, ideally, comparative evidence that model-guided decisions improve patient outcomes over standard care. AI systems use computational algorithms to identify patterns in high-dimensional clinical, imaging, and sensor data. In stroke research, these methods have been evaluated for prognostic estimation, treatment-decision support, and longitudinal recovery monitoring. Daidone et al. [39] described how ML models can represent nonlinear relationships among heterogeneous clinical variables used for outcome prediction. Gupta [40] mapped AI-based neurorehabilitation technologies across diagnostic, prognostic, and implementation domains. These sources describe potential applications rather than establishing routine clinical effectiveness. Reviews by Lee et al. [41] and Chavva et al. [42] describe automated image analysis, lesion detection, segmentation, tissue-fate prediction, and the integration of neuroimaging with clinical data in acute stroke care. Such information may contribute to downstream planning by characterizing lesion burden, initial severity, and acute treatment, but these reviews do not provide primary evidence that an acute diagnostic model improves rehabilitation outcomes.

### Technical overview

ML and DL are related but distinct computational approaches. ML includes algorithms that learn predictive relationships from data, often using prespecified features. DL uses multilayer neural networks to learn representations directly from complex inputs such as images, audio, and longitudinal sensor signals. Both approaches may overfit small or selected datasets and therefore require appropriate internal validation followed by independent external and prospective evaluation. In poststroke prognostication and neurorehabilitation, ML has been applied to outcomes such as language, cognition, motor recovery, and functional independence. Apostolidis et al. [43] reviewed ML approaches for predicting language and cognitive rehabilitation outcomes after stroke. Such models may identify associations between baseline characteristics and later outcomes, but prediction alone does not establish that model-guided care improves recovery. DL, a subset of ML, has been investigated for complex data types such as images, audio, and longitudinal sensor streams. Yang et al. [44] conducted a systematic review and meta-analysis of 7 cohort studies of acute stroke prognosis and reported a pooled area under the receiver operating characteristic curve (AUC) of 0.872 (95% confidence interval [CI], 0.862 to 0.881). The reported subgroup AUCs were 0.888 (95% CI, 0.872 to 0.904) for DL, 0.852 (95% CI, 0.835 to 0.869) for logistic regression, 0.863 (95% CI, 0.845 to 0.882) for random forest, and 0.905 for the support vector machine and XGBoost subgroups; the latter estimates had wide CIs.

The study-level comparisons summarize acute prognostic discrimination, and the apparent differences between algorithms depend on the populations and validation methods included. They therefore provide no basis for selecting a clinically superior algorithm for rehabilitation planning. Fernandes et al. [45] synthesized 25 reviews of ML and DL applications in stroke diagnosis, principally classification, segmentation, and object detection. As a secondary synthesis, it offers a broad map of diagnostic research without providing prospective clinical validation or evidence of rehabilitation effectiveness. Small samples, limited transparency, and overfitting continue to impede clinical translation [46]. Campagnini et al. [47] identified similar problems in their review of ML methods for poststroke functional-recovery prediction. More interpretable models, robust validation, and privacy-preserving multicenter data strategies may address some of these limitations. Martínez-Cid et al. [48] described an explainable decision-support system for personalizing rehabilitation routines, although its effect on clinical care has yet to be evaluated prospectively. Their clinical value will depend on transparent reporting, validation in independent populations, and workable integration into rehabilitation services.

### AI algorithms and models

Fig. 5 compares 4 broad algorithm families used in stroke research. Convolutional neural networks (CNNs) analyze spatial patterns, recurrent neural networks (RNNs) and long short-term memory (LSTM) networks model sequential data, transformers use attention mechanisms to represent global relationships, and ensemble methods combine multiple learners for structured-data prediction.

**Fig. 5.**
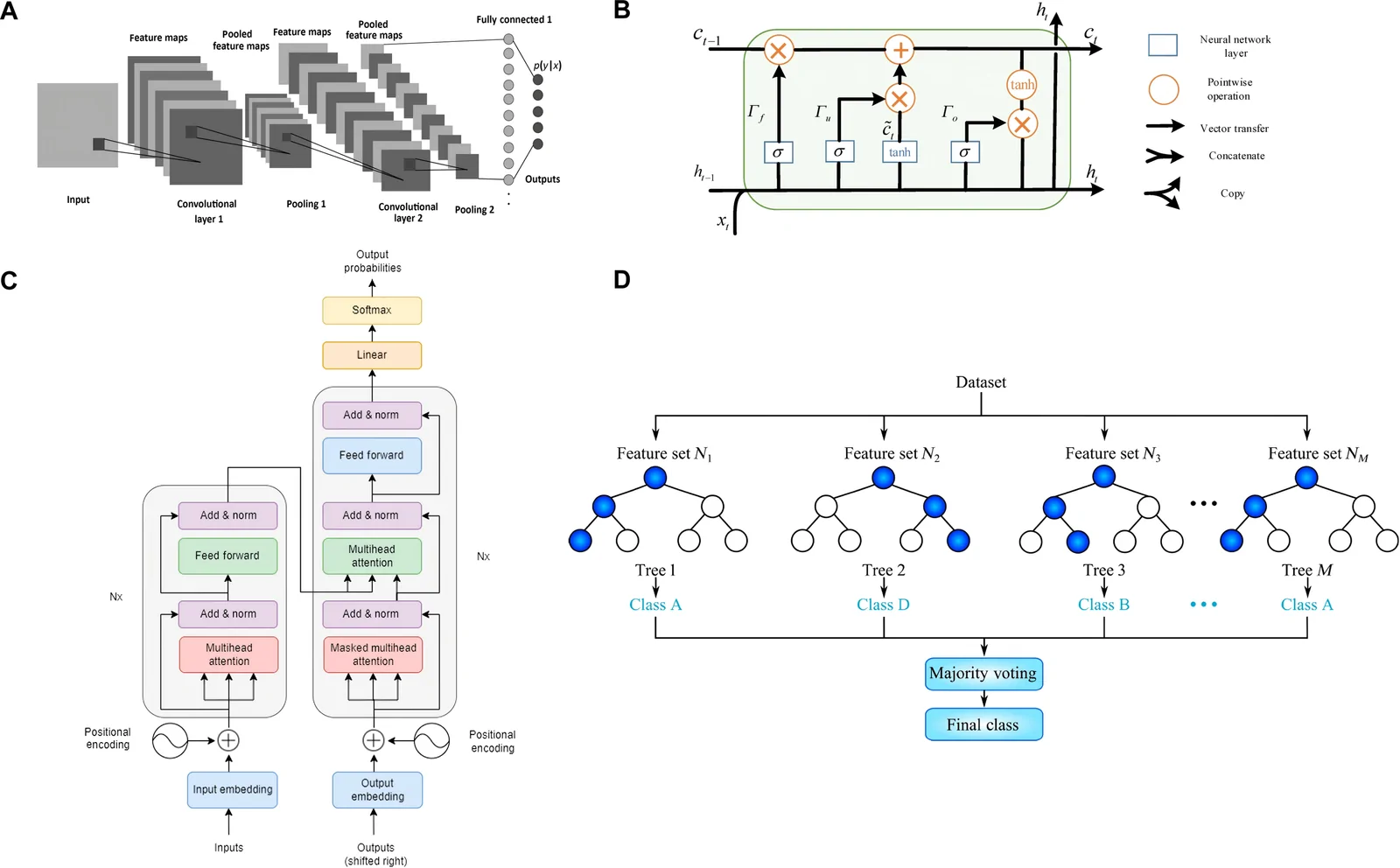
Core artificial intelligence algorithm architectures relevant to stroke rehabilitation. (A) Convolutional neural network architecture for hierarchical spatial-feature extraction [114]. (B) Recurrent neural network and long short-term memory architecture for sequential data [115]. (C) Transformer architecture using self-attention [116]. (D) Ensemble-learning approach for structured-data prediction [117]. Source: adapted conceptually from Refs. [114–117]; no performance comparison is implied.

Their practical suitability differs by clinical data type. CNNs are commonly applied to spatial tasks such as lesion detection, segmentation, and feature extraction from computed tomography (CT) or magnetic resonance images. RNNs and LSTMs can represent ordered observations such as repeated rehabilitation assessments, wearable-sensor streams, or exercise sequences. Transformers may be useful for longer temporal dependencies or multimodal combinations when sufficiently large datasets are available.

Random forests and gradient-boosting models are often practical for structured clinical, demographic, and rehabilitation-scale variables, whereas support vector machines may be useful for smaller, high-dimensional feature sets. Careful validation is required to limit overfitting. These task–algorithm correspondences indicate plausible use cases, not inherent clinical superiority; selection should be driven by the prediction target, sample size, input structure, interpretability requirements, and validation design.

For example, CNN–LSTM-based architectures and attention mechanisms have been used to derive gait phases and hip-angle estimates from electromyography (EMG) signals in a real-time lateral-walking setting [49]. Such results illustrate a technical use case for time-series movement analysis rather than evidence of stroke-specific clinical benefit.

Algorithm selection should reflect the clinical task, data structure, computational requirements, and balance between performance and interpretability. Support vector machines construct separating hyperplanes in transformed feature spaces, whereas decision-tree and random-forest methods model nonlinear interactions in structured data. Su et al. [50] applied support vector machines, random forests, gradient boosting, and deep neural networks to an acute-ischemic-stroke registry and used Shapley additive explanations to interpret predictions of discharge modified Rankin Scale and in-hospital deterioration. This acute early-outcome study did not evaluate rehabilitation progress or show that model use improved rehabilitation decisions.

Fig. 5A to C illustrate CNN, RNN/LSTM, and transformer architectures investigated in stroke assessment and rehabilitation. Their performance depends on data quality, sample size, task definition, and validation design. Zihni et al. [51] examined explainable prediction of functional outcomes, Lee [52] investigated hybrid systems that combine automated assessment with clinician oversight, and Lu and Qi [53] described an interactive rehabilitation system.

In a 2020 Association for Computing Machinery conference study of 3 upper-limb exercises, Lee et al. [54] reported that incorporating therapist feedback increased the mean F1-score from 0.8279 to 0.9116. This task-specific result supports technical feasibility in the study setting, but the report did not establish independent external validation or improvement in patient-level outcomes. Related studies proposed decision-support systems for rehabilitation data [55] and ML methods for assessing exercise quality [56].

Islam et al. [57] reported an explainable EEG-based model for stroke prediction. This work concerns stroke prediction from EEG signals and should not be interpreted as evidence that an AI-guided rehabilitation intervention improves functional recovery.

AI architectures have also been applied to physiological signals. Yu et al. [58] described an EMG-based system for analyzing muscle activity. Luvizutto et al. [59] reviewed poststroke assessment tools within the International Classification of Functioning, Disability and Health framework. A related system analyzed body-motion biosignals during walking [60]. These studies demonstrate approaches to sensor analysis, but they do not establish that real-time prediction improves rehabilitation outcomes.

Together, these algorithmic approaches may support stroke assessment and rehabilitation planning when matched appropriately to the clinical task and data type.

Many of the algorithms are technically established, but rehabilitation-specific validation and clinical impact studies are still uncommon.

## Applications of AI in Stroke Outcome Prediction

### Clinical task framework and acute-care context

Stroke prediction research includes acute diagnosis, acute prognostication, rehabilitation-outcome forecasting, and long-term risk management. Each addresses a different clinical question and requires different supporting evidence. Even technically sophisticated models may be difficult to use across heterogeneous settings because performance in a development dataset may not persist in other populations or fit routine workflows.

Acute imaging and early prognostic models provide baseline information that may inform subsequent rehabilitation planning. Neuroimaging can characterize lesion location, volume, and tissue viability; early clinical severity scores capture initial impairment; and acute-treatment data document reperfusion status and complication risk. These variables may be incorporated as covariates in rehabilitation prediction models, while timely acute diagnosis and treatment may influence the functional baseline from which rehabilitation begins.

Measures of acute diagnostic accuracy or early prognostic discrimination answer an early-care question; they do not show that a rehabilitation intervention is effective or that algorithm-guided adjustment improves long-term recovery. Rehabilitation claims therefore require models developed and validated for outcomes such as motor function, ADLs, participation, or quality of life. Whenever possible, studies should also test whether using the model changes clinical decisions or patient outcomes compared with standard care.

Mainali et al. [61] reviewed ML in stroke diagnosis and outcome prediction, drawing particular attention to small datasets and continued dependence on expert oversight. Zhao et al. [62] examined neuroimaging, ML, and DL approaches relevant to ischemic stroke rehabilitation. Together, these reviews help define potential applications and recurring methodological gaps, but they are not primary validation studies. Because the cited literature spans several clinical tasks, its findings should be interpreted separately rather than combined into a claim for a single clinically validated rehabilitation tool.

The primary studies summarized in Fig. 6 illustrate the diversity of prediction targets, data sources, and validation designs. Yang and Guo used 156 dynamic susceptibility contrast perfusion-weighted imaging examinations from 88 hospitalized patients to predict 90-d modified Rankin Scale outcomes with 10-fold cross-validation. This represents internal validation in a small, single-center acute imaging dataset [63]. Campagnini et al. [64] screened 278 poststroke rehabilitation patients, analyzed 273 after prespecified exclusions, and internally cross-validated models for modified Barthel Index class transition; Shapley additive explanations were used to interpret the contribution of clinical predictors.

**Fig. 6.**
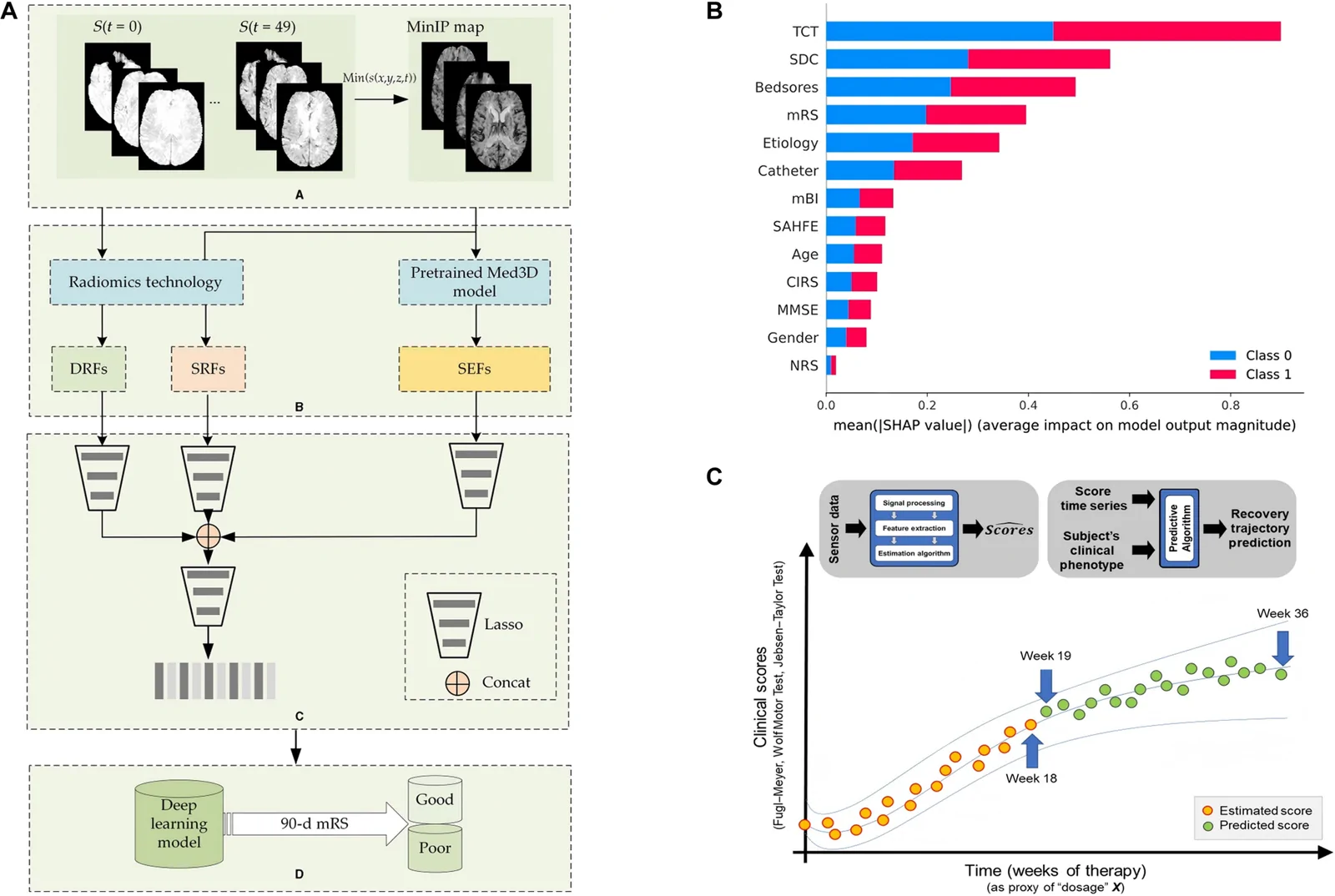
Diversity of artificial intelligence prediction frameworks relevant to stroke recovery. Three representative paradigms are shown: (A) whole-brain dynamic and static feature extraction from DSC-PWI for the prediction of 90-d ischemic stroke outcome [63], (B) clinical-scale-based machine learning with SHAP interpretation for functional class transition after poststroke rehabilitation [64], and (C) wearable-sensor estimation of clinical motor scores and longitudinal recovery trajectories [65]. Sources by panel: Refs. [63–65], respectively. The panels represent different targets and validation designs and should not be interpreted as equivalent evidence of rehabilitation efficacy. Letters within panel (A) denote workflow stages, not manuscript panel labels. CIRS, Cumulative Illness Rating Scale; DRFs, dynamic radiomics features; DSC-PWI, dynamic susceptibility contrast perfusion-weighted imaging; mBI, modified Barthel Index; MinIP, minimum intensity projection; MMSE, Mini-Mental State Examination; mRS, modified Rankin Scale; NRS, Numerical Rating Scale; SAHFE, Standardised Audit of Hip Fracture in Europe; SDC, Communication Disability Scale; SEFs, static encoding features; SHAP, Shapley additive explanations; SRFs, static radiomics features; TCT, Trunk Control Test.

Adans-Dester et al. [65] prospectively enrolled 37 people with acquired brain injury, including 16 stroke survivors, and used leave-one-subject-out cross-validation to estimate clinical motor scores from wearable-sensor data collected during structured tasks. Amador et al. [66] developed a multimodal model combining 4-dimensional CT perfusion imaging with clinical metadata to predict lesion and functional outcomes. Because that article was published after the March 2025 search cutoff, it is shown only as a postsearch contextual update and was not included in the 108-study synthesis.

Other relevant frameworks not depicted in Fig. 6 include Jabal et al.’s [67] interpretable prediction of 90-d outcome after thrombectomy using CT and CT angiography features from 293 patients at 2 centers and White et al.’s [68] magnetic resonance imaging (MRI) and tabular-data classification using 758 participants from the Predicting Language Outcome and Recovery After Stroke (PLORAS) resource. These studies address acute prognosis, score estimation, or symptom classification rather than demonstrated improvement in rehabilitation treatment.

Beyond acute imaging, studies have compared computational approaches for rehabilitation-relevant endpoints, but the clinical target and validation design must remain explicit. Campagnini et al. [64] used data from 278 poststroke rehabilitation patients and internally cross-validated models for modified Barthel Index class transition. Scrutinio et al. [69] analyzed 1,207 patients with severe disability who had completed rehabilitation to predict 3-year mortality; this represents postrehabilitation risk stratification rather than prediction of treatment response.

Khorsand et al. [70] analyzed 457 patients treated with alteplase to predict acute-treatment outcome. The available report did not clearly establish independent external or prospective validation, and the task is not equivalent to rehabilitation recovery prediction. Results from these different endpoints cannot be combined conceptually as evidence that a particular algorithm is superior for rehabilitation.

Shafaat et al. [71] reviewed AI applications in ischemic stroke imaging, including automated infarct detection, Alberta Stroke Program Early CT Score (ASPECTS) calculation, segmentation, prognosis, and treatment selection. Zeleňák et al. [72] reviewed technologies across the acute stroke pathway, including prehospital care, diagnosis, treatment, and rehabilitation. Both are review articles rather than prospective validation studies. Their acute-care content is relevant to rehabilitation only indirectly, because earlier diagnosis, treatment selection, and baseline characterization can influence later recovery planning; neither source demonstrates that an imaging AI system improves rehabilitation efficacy.

Computational imaging and early prognostic models can add baseline information to subsequent care planning. Rehabilitation-specific models, however, need evaluation against functional, participation, or patient-reported outcomes measured during or after rehabilitation. The evidence summarized here is mainly exploratory or internally validated, leaving transportability, workflow performance, and clinical impact to be tested in independent and prospective studies.

### Rehabilitation-outcome prediction and longitudinal monitoring

AI prediction in stroke rehabilitation is still constrained by static outcome definitions, incomplete temporal modeling, small or selected cohorts, and sparse external validation. Cacciotti et al. [73] enrolled 127 patients with subacute ischemic stroke and 90 matched controls, used baseline EEG functional-connectivity measures, and assessed National Institutes of Health Stroke Scale (NIHSS) change at 40 d. The study included stroke classification, affected-hemisphere classification, and short-term functional-recovery prediction, but the available report did not clearly describe validation in an independent cohort. Halloran et al. [74] presented wearable-accelerometer remote monitoring as a conference paper, and Li et al. [75] described an Internet of Things architecture for stroke prevention, nursing, and rehabilitation management. Both sources primarily demonstrate monitoring or management systems; whether these systems improve rehabilitation outcomes has not been tested prospectively.

White et al. [68] used MRI and tabular data from 758 PLORAS participants to predict whether poststroke spoken-picture-description performance was in the aphasic range. A prespecified fifth matched group served as a held-out test set, but it was drawn from the same study resource and did not constitute prospective clinical impact validation. Kuźnar and Lorenc [76] studied only 20 patients who completed a 4-week home-rehabilitation program and developed models using the Rivermead Motor Assessment, ADL indicators, and demographic variables. This small exploratory study cannot establish transportability, treatment benefit, or readiness for routine care.

Chen et al. [77] retrospectively analyzed data from 124 participants in previous chronic-stroke rehabilitation studies and predicted post-intervention improvement in patient-reported activity and participation outcomes using demographics, stroke characteristics, and baseline assessment scores. Gandolfi et al. [78] applied random forest and explainable AI methods to predict upper-limb outcomes after rehabilitation in patients with subacute stroke. Both studies address who may improve after rehabilitation; neither tests whether AI-guided treatment produces additional recovery. Model outputs should therefore not guide treatment intensity or adjustment until they have undergone independent prospective validation and clinical impact evaluation.

### Acute intracerebral hemorrhage prognostication in the context of rehabilitation planning

Clinical evaluations of AI prediction models frequently use retrospective or selected cohorts, heterogeneous outcome definitions, and limited validation. These design features can reduce generalizability and may produce optimistic performance estimates when study populations do not represent routine stroke care.

Chen et al. [79] developed 2 CNN-based models to predict poor neurological outcome at hospital discharge, defined as a Glasgow Outcome Scale score of 1 to 3, in a multicenter retrospective cohort of 1,990 patients with intracerebral hemorrhage (ICH). The authors reported a training set of 1,599 patients and a test set of 391 patients and described nested 5-fold cross-validation. On the reported test set, the clinic–imaging fusion model achieved an AUC of 0.903, compared with 0.886 for the imaging-based model, 0.777 for the original ICH score, and 0.747 for the ICH grading scale (Table 2). These results quantify short-term discrimination within the reported retrospective dataset; they do not constitute independent cross-institutional external validation or evidence that model use improves emergency or rehabilitation outcomes.

**Table 2.** Area under the receiver operating characteristic curve for 4 models predicting poor neurological outcome at discharge in the multicenter retrospective study by Chen et al. [79]

Model	AUC on the reported test set
Clinic–imaging fusion model	0.903
Imaging-based model	0.886
Original ICH score	0.777
ICH grading scale	0.747

Note: AUC, area under the receiver operating characteristic curve; ICH, intracerebral hemorrhage. Chen et al. conducted a multicenter retrospective study involving 1,990 patients, comprising a training set of 1,599 patients and a test set of 391 patients, with nested 5-fold cross-validation. Poor neurological outcome at discharge was defined as a Glasgow Outcome Scale score of 1 to 3. Only the AUC values reported in the indexed article abstract are presented; other performance measures were not reproduced. Data derived from Chen et al. [79].

The reported AUCs describe discrimination within a retrospective development and test dataset rather than transportability across institutions or improvement in clinical outcomes. Emergency use would require independent external and prospective validation, reliable data integration, calibration assessment, workflow evaluation, and evidence that the predictions improve decisions or patient outcomes. Because Chen et al. [79] evaluated short-term status at discharge, this study should be interpreted as acute prognostic evidence rather than direct evidence of rehabilitation recovery. If independently validated, discharge-risk estimates could contribute to rehabilitation stratification, anticipated care intensity, and resource planning when interpreted alongside functional status, comorbidities, patient goals, and clinician assessment. They should not be used alone to determine access to rehabilitation or treatment intensity.

Liao et al. [80] used data from 132 people with chronic stroke to predict improvement in health-related quality of life after sensorimotor rehabilitation. Model development used a training/test split with 10-fold cross-validation in the training data; therefore, the findings remain internally validated within the study dataset. Pitchai et al. [81] used synthetic data to train an EMG-based stroke prediction system, but the article was subsequently retracted. It is therefore not used in this review as evidence of diagnostic validity, rehabilitation utility, or clinical maturity. Across neuroimaging, functional connectivity, ADL recovery, and quality-of-life prediction, performance must be interpreted alongside outcome definitions, follow-up, validation strategy, workflow compatibility, and whether model use changes clinical decisions.

Most prediction studies are retrospective and rely on internal or single-center temporal validation. Independent multisite evaluation and prospective studies of clinical impact are uncommon.

## Applications of AI in Stroke Rehabilitation Management

### AI-supported personalized rehabilitation planning

Many systems described as personalized still rely on group-level models. In practice, personalization often consists of adjusting treatment intensity, task difficulty, or feedback frequency within an existing protocol. Few studies model individual recovery mechanisms or incorporate the psychosocial factors that may influence treatment response.

Relevant sources of heterogeneity include lesion characteristics, age, comorbidities, baseline function, cognitive reserve, mood, family support, and access to therapy. Current models generally include only a subset of these factors, limiting the extent to which their recommendations can be considered individually tailored.

Deole and Dharwadkar [82] used clinical, behavioral, and rehabilitation variables in ML analyses of recovery-related patterns. Alignment with established clinical guidance may help ensure that algorithmic recommendations remain consistent with evidence-based rehabilitation practice [83]. However, retrospective associations do not establish causal treatment effects or show that algorithm-guided adjustment improves recovery.

AI models can process heterogeneous data, but rehabilitation datasets often contain missing values, inconsistent assessment schedules, and selection bias. Integrating sensor, wearable, and clinical-record data also creates challenges in temporal alignment and interpretation. If these limitations are not addressed during model development, the resulting associations may be misleading.

Real-time feedback and dynamic adjustment raise practical questions about clinical responsibility, the communication of recommendations, and whether frequent algorithmic changes improve outcomes compared with consistent progressive training. Most personalized rehabilitation studies remain exploratory, retrospective, or small. Large prospective studies are required before AI-generated rehabilitation plans can be recommended for routine care.

### AI-assisted rehabilitation technologies and candidate populations

Clinical evidence has not kept pace with the development of AI-assisted rehabilitation technologies. VR, robotic devices, and BCIs differ in how they deliver training, the motor and cognitive abilities they require, their technical complexity, and the level of supervision involved. The published literature focuses mainly on feasibility, short-term surrogate outcomes, and within-study comparisons. Independent validation, durable functional benefit, and superiority over standard care have seldom been examined.

VR applications use interactive feedback and simulated daily activities to support task-oriented practice, with some studies also exploring integration with BCIs [16–18,84]. Most reports involve small samples and short follow-up, so comparative durability relative to conventional task-oriented therapy remains uncertain. Successful participation requires comprehension of the task, sustained attention, adequate sensory capacity, and tolerance of the interface. These are practical selection considerations, not established clinical indications.

Robot-assisted therapy provides repetitive motor practice with measurable, adjustable assistance. Systems have been developed for upper-limb movement [19–24], gait and lower-limb training [25], and AI-assisted therapy protocols [26]. They may be considered for patients who need movement assistance or a standardized practice dose, although the affected limb, residual movement, muscle tone, device fit, and available supervision all influence feasibility. At present, these devices are therapeutic adjuncts, and greater precision or training volume should not be taken as evidence of long-term functional benefit.

Recent work on hip-exoskeleton control has also addressed continuous locomotion-mode perception across level-ground and stair walking; however, the reported evaluation involved 7 participants and did not test poststroke rehabilitation outcomes [85].

BCIs translate motor imagery or other neural signals into feedback or assisted movement. In the study by Irimia et al. [27], 5 stroke patients completed 10 to 24 sessions and achieved a mean within-study EEG classification accuracy of 87.4%. The result was not externally validated, and motor recovery or superiority over standard rehabilitation was not demonstrated. Other exploratory applications include soft-glove control and cognitive rehabilitation [28,29]. BCIs may be considered when voluntary movement is very limited, provided that patients can understand and sustain the task and that usable neural signals, adherence, and specialized technical support are available.

Recent EEG-transformer work in steady-state visually evoked potential BCI further illustrates how data augmentation and temporal–spatial feature learning may improve neural-signal decoding on public datasets [86]. These algorithmic results should not be interpreted as validation of poststroke functional recovery or clinical effectiveness.

A representative BCI workflow is summarized in Fig. 7.

**Fig. 7.**
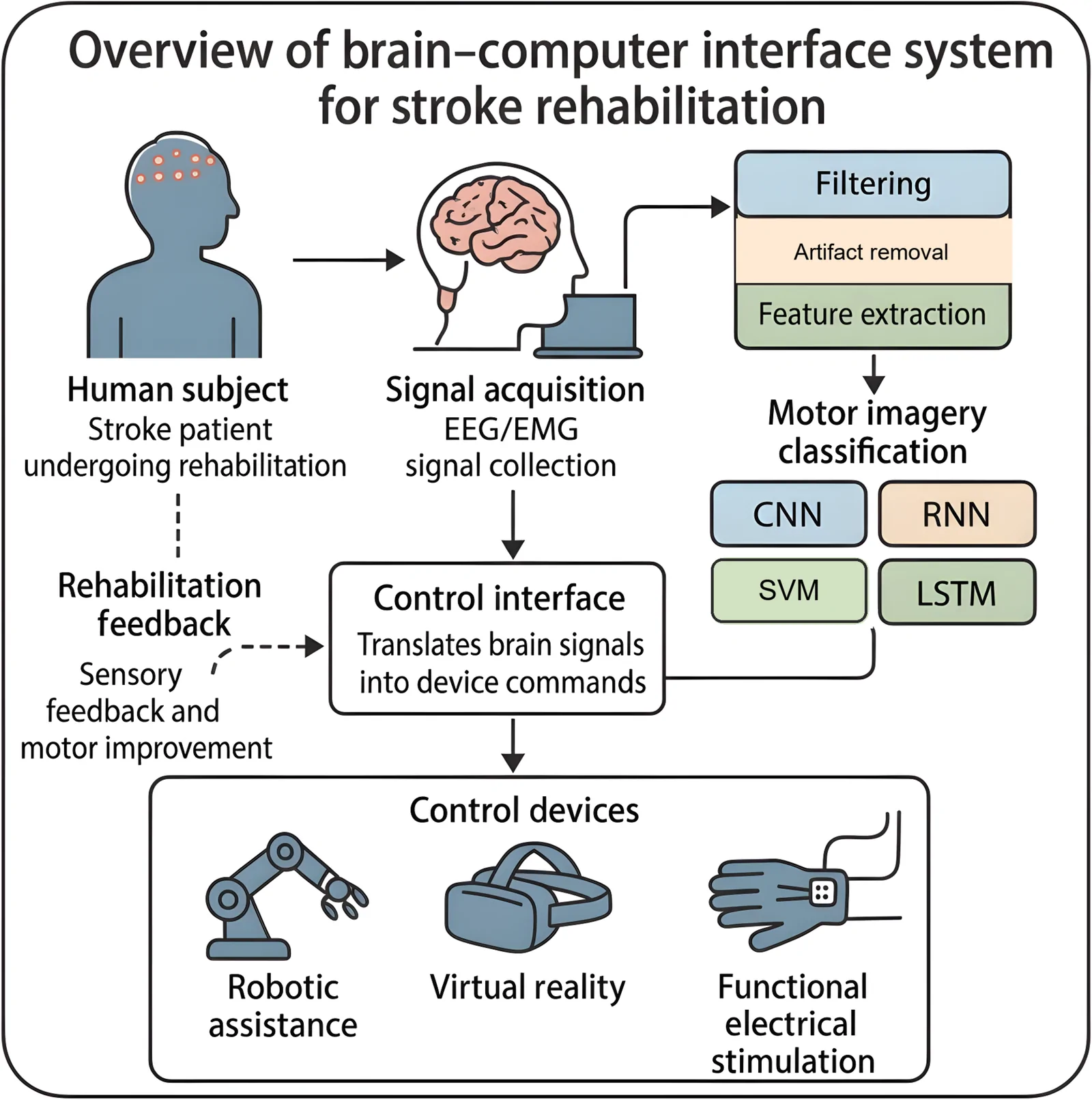
Brain–computer interface workflow for stroke rehabilitation. The diagram summarizes neural-signal acquisition, signal preprocessing, motor-imagery classification, control-interface translation, rehabilitation feedback, and assisted task practice. Source: conceptual figure created by the authors from the studies cited in the “AI-assisted rehabilitation technologies and candidate populations” section. CNN, convolutional neural network; EEG, electroencephalography; EMG, electromyography; LSTM, long short-term memory; RNN, recurrent neural network; SVM, support vector machine.

In practical terms, VR places the greatest emphasis on active interaction, robotic systems on assisted repetitive movement, and BCIs on neural-signal tasks that may remain possible despite very limited voluntary movement. Matching a patient to a technology also requires attention to motor, cognitive, sensory, and fatigue profiles, as well as device tolerance and rehabilitation goals. Combined systems are technically possible, but their clinical value still needs prospective evaluation. These considerations are intended to guide candidate selection and should not be read as formal indications.

Remote monitoring extends this technology spectrum through gamified telerehabilitation, wearable assessment, intelligent footwear, and posture monitoring [87–92]. These applications may support follow-up outside specialist centers, but current evidence is mainly observational or single arm. Prospective studies should evaluate alert burden, workflow integration, data security, and clinically meaningful outcomes.

### Clinical applications and evidence

Clinical studies of AI-assisted stroke rehabilitation frequently use small or selected samples, surrogate technical endpoints, and limited validation, which restricts generalizability. Weiss Cohen and Regazzoni [93] described a leap-motion-controller-based hand-rehabilitation assessment system that used supervised ML to characterize movement patterns. This system-development evidence supports the technical feasibility of automated movement assessment but does not by itself establish longitudinal recovery prediction, comparative therapeutic effectiveness, or clinical impact. Quantitative comparisons across accelerometer and motion-tracking studies require a traceable common source, consistent definitions of the predicted outcome, and harmonized reporting of sample size, monitoring duration, and validation design.

Deole and Dharwadkar [82] also associated lifestyle factors and stroke subtype with recovery-related outcomes in a limited retrospective dataset. These associations require prospective longitudinal validation before they can support real-time rehabilitation decisions.

Across AI-assisted planning, therapeutic devices, remote monitoring, and movement assessment, the literature consists mainly of exploratory, pilot, and early clinical studies. Comparative-effectiveness and implementation evidence is limited.

Experience with pattern-recognition-controlled myoelectric prostheses also illustrates a broader translational caution: increased device functionality and task repertoire may coexist with reduced control reliability or limited improvement in everyday activities [94].

## AI Challenges and Bottlenecks in Stroke Rehabilitation

### Data-related challenges

#### Data quality and heterogeneity

Model performance depends on representative training data. Stroke-rehabilitation datasets vary in lesion characteristics, comorbidities, treatment exposure, assessment timing, and recovery trajectory. Zu et al. [95] identified heterogeneity and selection bias as barriers to generalizability. Large datasets may support model development, but clinically meaningful features remain difficult to extract from noisy and multimodal records [96]. Standardized data definitions, preprocessing, and external validation are therefore essential.

#### Data privacy and security

Rehabilitation systems may process imaging, physiological signals, gait measurements, and home-monitoring records. Governance should specify what is collected, who can access the data, how long records are retained, and whether data can be reused for model development.

Senadheera et al. [7] identified privacy and security as major barriers to wider adoption of AI in stroke rehabilitation. Cross-institutional data sharing is also constrained by differences in governance, consent, and data-protection requirements, including the General Data Protection Regulation. These constraints can limit the development of sufficiently large and diverse multicenter datasets.

### Model-related challenges

#### Model transparency and interpretability

The “black box” problem remains a barrier to clinical translation. Ono et al. [97] developed a model for walking independence at discharge using admission data from 280 patients in a recovery-phase rehabilitation ward and evaluated it in 134 patients admitted during a later period. In the temporal validation cohort, the reported AUC was 0.917, with accuracy of 79.9%, sensitivity of 95.7%, specificity of 62.5%, positive predictive value of 73.6%, and negative predictive value of 93.5%. A later cohort provides stronger evidence than a random split, but this remains single-institution temporal validation rather than geographic external validation or a clinical impact study. The results support prognostic discrimination within that setting, not proof that model use improves rehabilitation decisions or outcomes.

In stroke rehabilitation, clinicians must understand the basis of model outputs before incorporating them into individualized treatment plans. Amann [98] argued that transparency influences both clinician acceptance and patient confidence in ML-supported risk prediction and prevention. Explanations should therefore identify the variables that drive a recommendation and clarify the conditions under which the model may fail.

#### Model reliability and generalization ability

Overfitting is common when models are trained on small or narrowly selected cohorts. Bo et al. [99] proposed a progressive model for home-based rehabilitation, but its transportability beyond the development setting remains uncertain. Reliability requires diverse datasets, independent external validation, calibration assessment, and testing in clinically heterogeneous populations.

The progressive home-rehabilitation prediction framework discussed above is illustrated in Fig. 8.

**Fig. 8.**
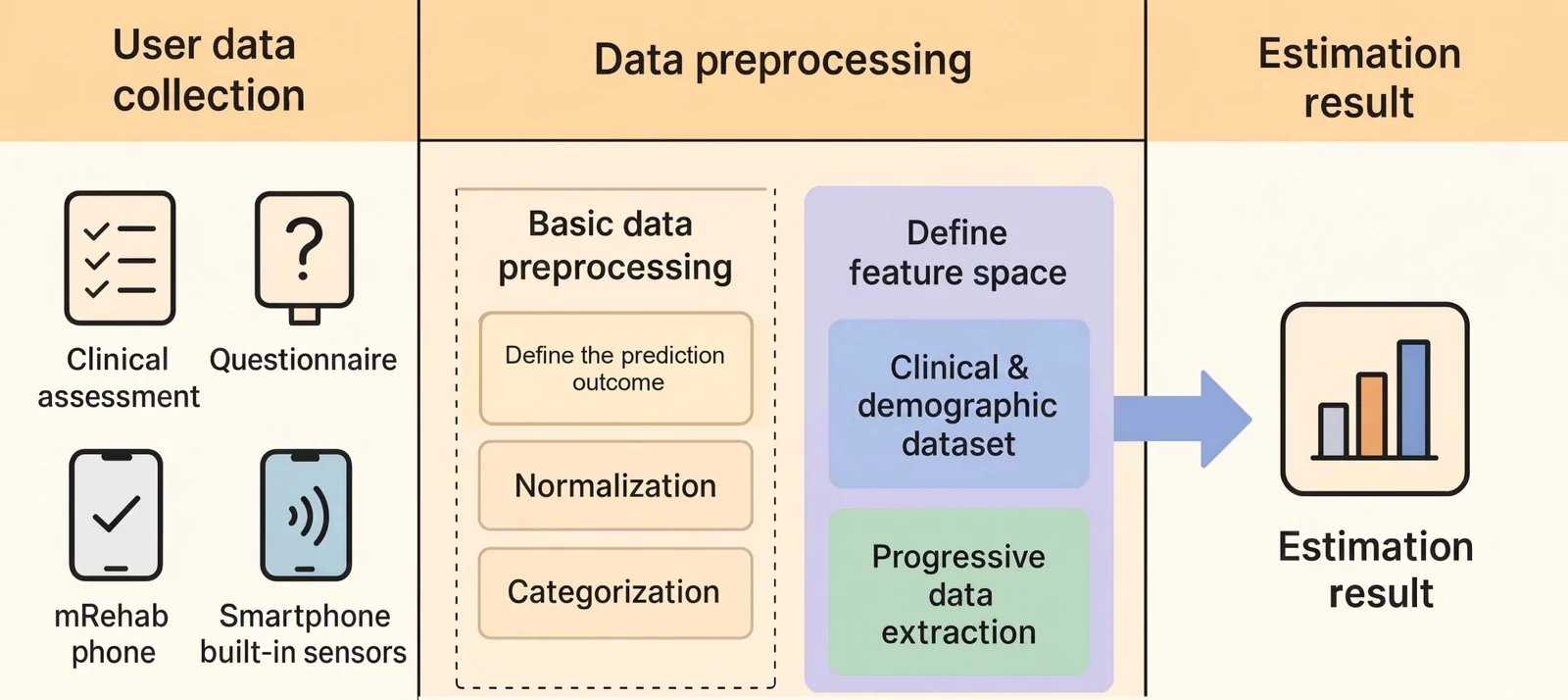
Progressive prediction framework for home-based stroke rehabilitation. Clinical assessments, questionnaires, and smartphone sensor data undergo basic preprocessing and progressive feature extraction before estimation of the rehabilitation outcome. Source: adapted conceptually from Bo et al. [99]; the figure does not imply external validation, transportability, or clinical effectiveness.

Bo et al. [99] retrospectively analyzed a 6-week home-based mobile rehabilitation program involving 16 stroke survivors and 12 rehabilitation activities. The authors combined clinical and demographic variables with movement-phenotyping data and compared multiple linear regression with random forest. Using data from the first 2 weeks, they reported root mean square error (RMSE) values of 0.1050 for multiple linear regression and 0.0011 for random forest. Because RMSE is scale dependent, these values cannot be compared across studies that use different outcome scales or preprocessing methods.

The report did not describe validation in an independent external cohort. The findings should therefore be classified as exploratory within-dataset performance rather than evidence of generalizability or clinical effectiveness. The progressive use of 2-, 4-, and 6-week data suggests a possible approach to updating predictions over time, but prospective evaluation in larger and more diverse home-rehabilitation populations is required.

Vodencarevic et al. [100] used registry data from 384 patients with ischemic stroke and double-nested cross-validation to predict 1-year recurrence, reporting an internally validated AUC of 0.70 (95% CI, 0.64 to 0.76). This task concerns recurrent-stroke risk rather than rehabilitation response, and the study did not provide independent external validation or clinical impact evidence. The result supports further evaluation for follow-up risk stratification but does not establish clinical effectiveness. More broadly, model generalizability depends on whether validation populations represent the heterogeneity of routine rehabilitation practice.

### Limitations of clinical application

Clinical operability and trust are central implementation concerns. AI may support rehabilitation counseling, monitoring, and vocational services, but it cannot fully represent motivation, mood, family support, or the therapeutic relationship [101]. Clinicians and patients may therefore be reluctant to use recommendations that are poorly explained or difficult to integrate into routine care [102].

In a brief *Nature Reviews Neurology* commentary rather than an original validation study, Leslie-Mazwi and Lev [103] discussed the potential clinical role of AI in large-vessel-occlusion stroke care. This source can support discussion of implementation opportunities and barriers, but it should not be used as primary evidence for sensitivity, specificity, workflow benefit, or patient outcomes. Acute imaging tools are relevant to rehabilitation planning only indirectly through earlier diagnosis, treatment, and baseline characterization; their diagnostic performance does not establish later rehabilitation benefit.

Clinical deployment therefore requires both technical validation and implementation planning. Models should provide explanations appropriate to the clinical task, identify important failure modes, and support communication between clinicians and patients. Interpretability alone is insufficient, but opaque recommendations are unlikely to be trusted or used safely.

#### Compatibility with conventional clinical practice

AI should support rather than replace established rehabilitation care. New technologies must fit multidisciplinary workflows and protocols [25]. Appropriate functions may include progress monitoring, change detection, and technology-supported task practice.

AI-supported recommendations should also be understandable to patients and families. When a system proposes a change in training intensity or task selection, clinicians should be able to explain the rationale, uncertainty, and available alternatives.

The intended compatibility between AI decision support and conventional rehabilitation practice is summarized in Fig. 9.

**Fig. 9.**
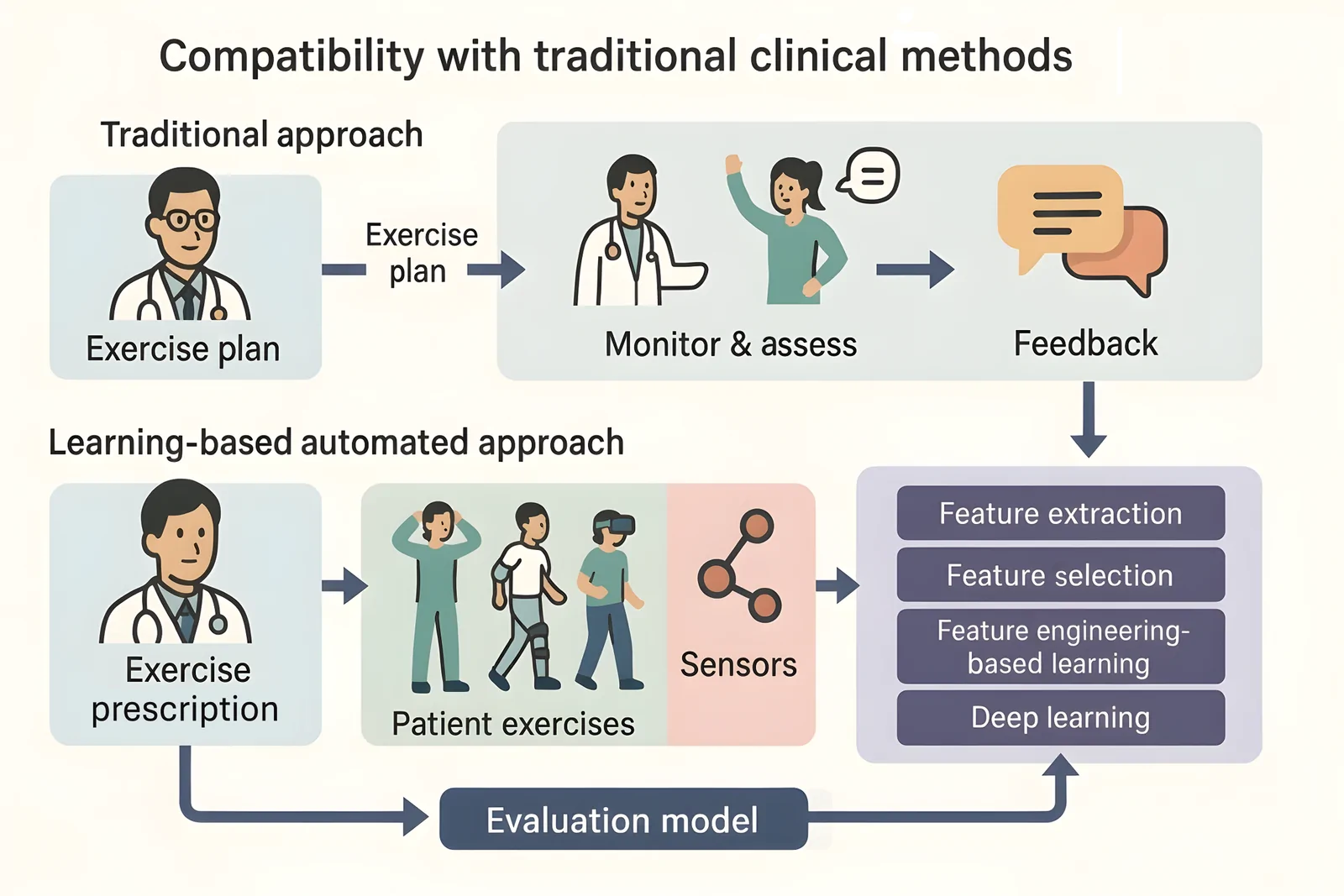
Integration of artificial intelligence decision support with conventional rehabilitation practice. The upper pathway shows conventional exercise planning, clinician monitoring, and feedback. The lower pathway shows how sensor-derived features and a learning-based evaluation model may supplement this workflow. Source: conceptual figure created by the authors.

Data, interpretability, and workflow barriers are well described, but few studies have tested whether proposed solutions improve implementation, safety, or equity in practice.

## Future Development Directions and Trends

### Potential for personalized treatment in stroke rehabilitation

Many current AI systems define personalization narrowly by adjusting training parameters or risk estimates using demographic, clinical, or imaging variables. They rarely represent the full range of factors that shape recovery, including cognition, mood, family support, the home environment, and potential for neural adaptation. Li [104] described the proposed use of clinical data, neuroimaging, and motor assessments to generate individualized protocols. Such approaches primarily identify associations in existing data rather than mechanisms of recovery. Claims of improvement over standardized protocols must therefore be evaluated in relation to outcome definitions, comparators, cost, and validation design.

Future personalization research should determine whether model-guided changes improve patient-centered outcomes rather than merely altering protocol parameters [105].

### Interdisciplinary technology integration

Future integration should focus on closed-loop systems in which EEG, EMG, wearable sensors, or other patient signals inform robotic assistance, extended-reality tasks, and adaptive feedback [6,106–109]. The purpose of integration should be to match assistance and task demands to the patient’s changing functional state, not simply to combine multiple devices. Development priorities include interoperability, usability, cost, clinical supervision, and prospective comparison with standard rehabilitation.

Multimodal neurophysiological assessment may also help characterize rehabilitation-related network changes. In Parkinson’s disease, combined EEG and functional MRI markers were associated with motor-rehabilitation responsiveness in a 52-participant cohort [110]. Whether analogous markers can guide stroke rehabilitation requires stroke-specific prospective validation.

Technology-intensive systems should also be evaluated against lower-cost approaches rather than in isolation. Tai Chi and acupuncture have been examined in the stroke-rehabilitation literature [111–113], although these sources represent different intervention types and levels of evidence and should not be treated as direct equivalents to AI-assisted technologies.

### Intelligent remote monitoring and home rehabilitation

Wearable devices and remote-monitoring systems may extend rehabilitation assessment into the home by collecting activity, movement, or physiological data [107]. Automated analysis can support feedback and clinician review, but treatment changes should remain clinically supervised and should be evaluated against meaningful functional outcomes.

Reviews of AI-supported rehabilitation and telerehabilitation describe potential benefits for self-management and continuity of care [4,6]. However, outcomes vary across interventions and populations. Effectiveness depends not only on the technology but also on usability, motivation, cognitive capacity, caregiver support, connectivity, and integration with clinical services.

A representative remote-rehabilitation training system is shown in Fig. 10.

**Fig. 10.**
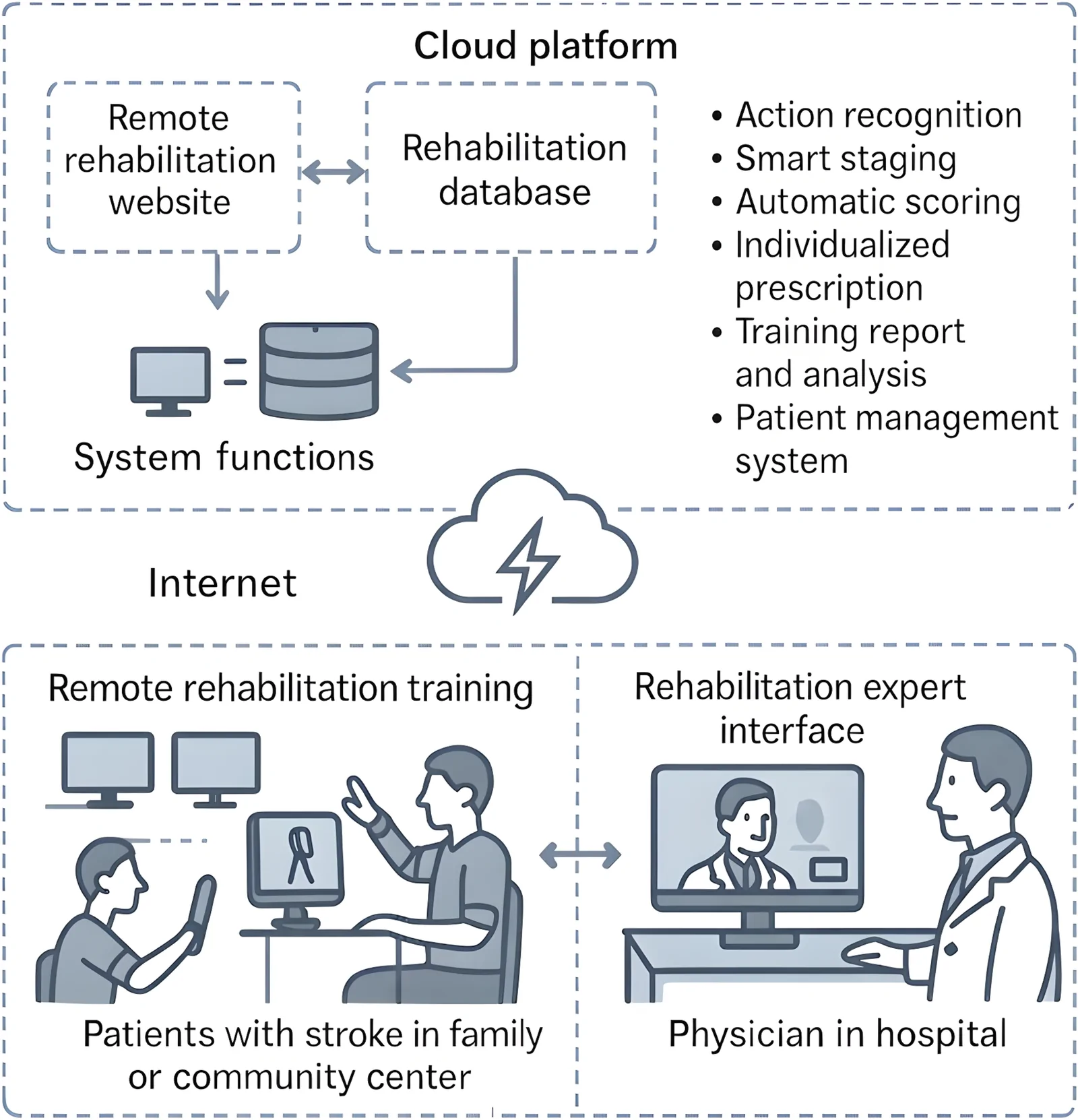
Remote rehabilitation training and monitoring system for people after stroke. The system connects home- or community-based training with a remote rehabilitation website, rehabilitation database, cloud-based analysis functions, and a hospital-based expert interface. Source: conceptual figure created by the authors from the remote-rehabilitation studies cited in the “Intelligent remote monitoring and home rehabilitation” section.

The economic value of remote rehabilitation remains uncertain. Home-based services may reduce travel, but they require devices, technical support, data infrastructure, and clinician oversight. These costs should be compared with conventional rehabilitation in formal economic evaluations. Remote care may also widen access gaps for people with limited connectivity, digital literacy, cognitive capacity, or home support. Internet of Things systems introduce additional concerns about data security, device reliability, and clinical governance that should be addressed before large-scale deployment.

### Data privacy, security, and governance standards

Privacy and security in AI-assisted rehabilitation extend beyond data encryption. Rehabilitation systems may collect imaging, movement, physiological, and home-monitoring data. Patients should be informed about what is collected, who can access it, how long it is retained, and whether it may be reused for model development. Senadheera et al. [7] identified privacy and security as major barriers to wider adoption of AI in stroke recovery and rehabilitation.

Future systems require clear governance, audit trails, consent procedures, and rules for cross-institutional or cross-border data sharing. Technical safeguards and regulatory standards should be developed together rather than treated as separate issues [105].

### Interpretability and clinical adaptability of AI models

The “black box” problem is a practical barrier because clinicians must explain treatment decisions to patients and families. Post hoc explanation tools may clarify individual predictions, but they do not establish how a model will perform outside its development population.

Ono et al. [97] reported favorable discrimination in a temporally separated cohort from the same rehabilitation setting, but such performance does not remove the need for transparency, geographic validation, calibration assessment, and clinical impact evaluation. There is also a trade-off between model complexity and ease of explanation. More complex models may improve selected prediction metrics but can be harder to audit, reproduce, and explain at the bedside.

Explanation requirements differ by task. Model evaluation requires information about overall behavior and failure modes, whereas individual care requires patient-level explanations of the variables contributing to a recommendation.

Clinical adaptability also depends on how AI recommendations interact with clinician judgment. Models trained on curated research datasets may perform less well in routine care when patient characteristics, outcome measures, or assessment schedules differ from those in the development cohort.

Cross-technology integration is still at an early stage. Its added complexity will be justified only if studies include appropriate patient stratification, usability testing, and prospective evidence of meaningful clinical benefit.

## Summary

Taken together, evidence maturity is uneven. Acute imaging and prognostic models are closest to potential use for early stratification, whereas rehabilitation-specific prediction and AI-enabled rehabilitation modalities remain supported mainly by internal validation or exploratory studies. The key translational gap is therefore not the availability of algorithms, but evidence that independently validated and calibrated systems improve rehabilitation decisions or patient-centered outcomes. Until such evidence is available, AI should be used only as an adjunct to multidisciplinary rehabilitation and interpreted alongside clinician assessment and patient goals.

### Limitations of this review

Selection and publication bias may have influenced this structured narrative review. The formal selection process covered 4 databases and included only English-language sources available through March 2025. Supplementary searching of reference lists and conference proceedings was not documented with sufficient detail for inclusion in the formal selection counts; consequently, those sources were used only for contextual discussion and were excluded from the 108-publication database-derived synthesis. Study designs, populations, outcome definitions, and validation strategies varied substantially, precluding meta-analysis and a uniform formal risk-of-bias assessment. Reason-specific counts for full-text exclusions were also unavailable. Table 1 supplies context from an external scoping review and does not represent the 108 publications synthesized here. Accordingly, the review maps research themes and evidence maturity; it does not provide pooled estimates of treatment efficacy or proof of clinical effectiveness.

## Data Availability

No new datasets were generated or analyzed in this study. The literature-search strategy and study-selection materials available for this review may be obtained from the corresponding authors upon reasonable request.
